# Demographics, Injury Patterns, Injury Severity and Injury Predictors in Children with Non-Fatal Injuries Due to Road Traffic Injuries: An Analysis by Mode of Transportation

**DOI:** 10.3390/children13050687

**Published:** 2026-05-16

**Authors:** Randall T. Loder, Hannah Koch

**Affiliations:** 1 Department of Orthopaedic Surgery and Riley Children’s Hospital, Indiana University School of Medicine, 705 Riley Hospital Drive, Suite 1100, Phase 1, Indianapolis, IN 46202, USA; 2 School of Medicine Student Affairs, Indiana University, Indianapolis, IN 46202, USA

**Keywords:** child, injury, road traffic, NEISS, diagnosis, fracture, emergency department

## Abstract

**Background/Objectives**: The purpose of this study was to analyze the demographics and injury patterns of children with transportation-related non-fatal injuries occurring on public roads, streets and highways using a nationwide emergency department (ED) database. **Methods**: Data from the National Electronic Injury Surveillance System (NEISS) All Injury Program (AIP) 2005–2021 was used. Five transportation methods (motor vehicle occupant, bicyclist, pedestrian, motorcyclist, other) occurring on a public highway, street, or road were analyzed. Statistical analyses were performed with SUDAAN 11.0.01™ software to obtain national estimates. **Results**: There were an estimated 8,188,810 ED visits for traffic-related injuries in children; the median age is 14.3 years. Sex distribution was equal; 93.4% were discharged from the ED, and the head/neck was the most injured area (51.9%). The most common diagnoses were contusion (35.7%), strain/sprain (28.0%), internal organ injuries (13.3%), fracture (8.4%), lacerations (7.4%) and concussions (4.1%). Predictor variable of not being discharged from the ED was the presence of a fracture (OR = 119.7 [71.3, 200.7], *p* < 0.0001), injury to the trunk (OR = 3.2 [2.7, 3.8], *p* < 0.0001), a pedestrian (OR = 3.9 [2.8, 5.3], *p* < 0.0001), those <1.5 years old (OR = 4.3 [2.8, 6.6], *p* < 0.001), and males (OR 1.5 [1.4, 1.6], *p* < 0.0001). The greatest prevalence of head/neck fractures was in motor vehicle occupants (23.3%), upper extremity fractures in bicyclists (73.1%) and motorcyclists (49.2%), and lower extremity fractures in pedestrians (56.6%). **Conclusions**: This detailed study can be used to compare/contrast these injuries to other countries regarding road traffic injuries in children. This data can be used to assess the outcomes of prevention strategies introduced in the future.

## 1. Introduction

Transportation is a fundamental need for society [1,2]. It has evolved from simple walking to more efficient methods (in terms of distance traveled per unit of time) [2]. While there are many different transportation methods in modern society (roads, railways, waterways, air), roads are most commonly used by the average person for routine travel. This is true both in the United States as well as most other countries, acknowledging that rail travel is popular in certain countries (e.g., many European countries, Japan, India).

Injuries sustained on roads are unfortunately too common and carry a significant burden of disease [3]; children are uniquely susceptible to these injuries. Worldwide, road traffic injuries are the leading cause of death [4] in children 5 to 14 years of age, “with a child or adolescent between the ages 0 and 19 dying due to road traffic injuries every two minutes—or 200,000 every year” [4]. In the US, injury is also the leading cause of death in children and adolescents (1 to 19 years of age) [5], with injuries due to motor vehicles being the most frequent cause of injury until 2020, when fatalities due to firearms outpaced those due to motor vehicle crashes [6]. For non-fatal injuries, the quality of life following road traffic injuries is significantly reduced compared to the general population norms [7], especially in children [4]. These road injuries can be due to various modes of transportation, including motor vehicles, motorcycles, bicycles, and walking.

It was the purpose of this study to analyze the demographics and injury patterns of children with transportation-related non-fatal injuries occurring on public roads, streets and highways in the United States using a nationwide emergency department (ED) database. We specifically wished to focus on differences between several different transportation types—motor vehicles, pedestrians, bicycles, and motorcycles. This has not been previously done in the US with a nationwide database and can be used as baseline data to assess the effect of future prevention strategies.

## 2. Materials and Methods

Data from the National Electronic Injury Surveillance System (NEISS) All Injury Program (AIP) 2005–2021 was used [8]. The NEISS AIP is a data set of ~65 US hospitals having at least six beds and an emergency department (ED). All ED visits due to an injury are entered and coded daily. There are five hospital strata, and they are stratified by size according to the annual number of ED visits (four hospitals—categorized as small, medium, large, and very large), along with a separate stratum for children’s hospitals. The data is weighted, with each hospital’s weight being the inverse of the probability of selection for the hospitals in each stratum. The data set consists of many demographic variables, including the patient’s sex, age, treatment date, diagnosis, anatomic location of injury, incident locale of injury, disposition from the ED, and cause of injury. Due to deidentification of the patient, it cannot be determined if some are repeat visits from a single patient.

The cause of injury (ICAUSE_C) is divided into 24 different groups. For the purposes of this study, the first five groups—the transportation-related groups—were used; ICAUSE_C of 1 is a motor vehicle occupant, 2 a motorcyclist, 3 a bicyclist, 4 a pedestrian, and 5 other types of transport. For the transportation-related groups, they are subclassified by traffic-relatedness into three subgroups. Group 1 (traffic-related) is when the injury occurs on a public highway, street, or road; group 2 (nontraffic-related) is when the injury occurs in any place other than a public highway, street, or road (e.g., parking lots, home driveways, golf courses, etc.); and group 3 is when the injury does not involve either group 1 or 2 (e.g., air transport, railways, watercraft) or when the mode of transport is unknown. Only group 1 was evaluated in this study.

The exact definitions of the ICAUSE_C transportation groups need to be clearly understood. The NEISS definitions [8], quoting from the coding manual, are:

**Motor vehicle occupant**: Injury to a driver or passenger of a motor vehicle caused by a collision, rollover, crash or some other event involving another vehicle, an object, or a pedestrian. This category includes occupants of cars, pickup trucks, vans, heavy transport vehicles, buses, and SUVs. Injuries to occupants of other types of vehicles such as ATVs, snowmobiles, and go-carts fall in the category of “other transport.” The motor vehicle occupant group is subclassified as driver, passenger, getting into/out of the vehicle, or other.

**Motorcyclist**: Injury to a driver or passenger of a motorcycle resulting from a collision, loss of control, crash, or some other event involving a vehicle, object, or pedestrian. This category includes drivers or passengers of motorcycles (classic style), sidecars, mopeds, motorized bicycles, and motor-powered scooters.

**Bicyclist**: Injury to a bicycle rider from a collision, loss of control, crash, or some other event involving a moving vehicle or pedestrian. This category includes riders of unicycles, bicycles, tricycles, and mountain bikes. This category does not include injuries unrelated to transport (moving), such as repairing a bicycle.

**Pedestrian (struck by or against a vehicle)**: Injury to a person involved in a collision, where the person was not at the time of the collision riding in or on a motor vehicle, railway train, motorcycle, bicycle, airplane, streetcar, animal-drawn vehicle, or other vehicle. This category includes persons struck by cars, pickup trucks, vans, heavy transport vehicles, buses, and SUVs. This category does not include persons struck by other vehicles such as motorcycles, trains, or bicycles; these cases fall in the category of “other transport.”

**Other transport**: Injury to a person boarding, alighting, or riding in or on all other transport vehicles involved in a collision or other event with another vehicle, pedestrian, or animal not described above. It includes railway, water, air, space, animal and animal-drawn conveyances (e.g., horseback riding), ATVs, battery-powered carts, ski lifts, and other cable cars not on rails.

Disposition from the ED was classified as discharged or not discharged (admitted, observed, transferred to another hospital for further care). The anatomic area of injury is classified as head/neck, trunk, upper extremity and lower extremity. The head/neck includes the skull, ear, eye, face, mouth, and neck; the trunk includes the upper and lower trunk; the upper extremity includes the shoulder, humerus/arm, elbow, forearm, wrist, hand and finger; and the lower trunk includes the hip, femur/thigh, knee, leg/tibia/fibula, ankle, foot, and toe.

Details of the NEISS AIP can be obtained at the Inter-University Consortium for Political and Social Research (ICPSR) (https://www.icpsr.umich.edu/web/ICPSR/series/198 (accessed on 23 December 2023)) as well as the NEISS AIP variable description manual [8]. The NEISS AIP data for the years 2005 through 2021 was downloaded and those patients having an ICAUSE_C code of 1 through 5 and a TRAFFIC code of 1 were extracted. Those <19 years of age comprised the final data set. Subgroups by age were created and were <1.5, 1.5- to 3-, 4- to 6-, 7- to 12-, 13- to 15-, and 16- to 18-years old. These ages roughly correspond to the developmental ages of preambulatory, toddler, preschool/kindergarten, elementary school, adolescent pre-driver’s license, and adolescent post-driver’s license. These are not the customary groups that many are acquainted with: <1 (babies), 1 to 3 (toddlers), 3 to 5 (preschool), 5 to 12 (grade school), and teen (12 to 18) years of age. Our categories were a priori and developmentally created for the question at hand, that of transportation-related injuries. The pedestrian group assumes that the child is ambulatory, and up to 18 months of age is considered a normal age for walking [9]; thus, the cutoff is 1.5 years for non-walkers. While all those 13 years and older are teenagers, in this study regarding transportation, the ability of a teenager to drive centers around when they can legally obtain a driver’s license. In order to capture differences in exposure/mechanism (e.g., pre- vs. post-licensure adolescents), we created the two teen age groups.

### Statistical Analysis

National estimates of injuries were obtained with SUDAAN 11.0.01™ software (RTI International, Research Triangle Park, NC, USA, 2013) in order to account for the stratified and weighted nature of the data. The estimated number (N) of injuries is calculated, along with 95% confidence intervals [CI] of the estimate. When the raw number of patients (n) is <20, the estimated number (N) becomes unstable and should be interpreted with caution (https://www.cpsc.gov/Research--Statistics/NEISS-Injury-Data/Explanation-Of-NEISS-Estimates-Obtained-Through-The-CPSC-Website (accessed on 23 March 2026)); thus, we report both the n and N. Similarly, other analyses (e.g., regression analyses, statistical tests of significance) were also performed with SUDAAN accounting for the strata and weighted data. Analyses between groups of continuous data were performed with the t-test (2 groups) or ANOVA (3 or more groups). Differences between groups of categorical data were analyzed by the χ^2^ test. For the analyses of not being discharged from the ED as well as the occurrence of a fracture, variables having a *p* < 0.05 from bivariate analyses were further studied using univariable logistic regression analyses. Statistical significance was set at a *p*-value < 0.05.

## 3. Results

There were an estimated 8,188,810 [7,584,758–8,768,157] ED visits during the study period for traffic-related injuries in children (Table 1); the estimated number of all injury visits over this time period was 501,289,192.

**Figure 1:**
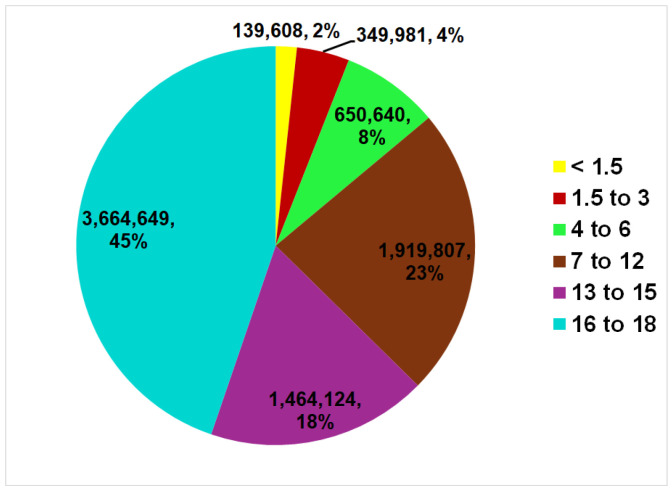
Age distribution of children sustaining traffic-related injuries seen in US EDs.

The average age and median age were 13.0 years and 14.3 years; the majority were 16 to 18 years old (44.8%) (Figure 1). Sex distribution was equal between males and females; 93.4% were discharged from the ED. From here forward, the tables only give the estimated value (N); the interested reader can find the n, N as well as the 95% confidence intervals of N in the Supplemental Files.

Sex distribution was equal between males and females; 93.4% were discharged from the ED. The head/neck was the most injured area (51.9%), and the two most common diagnoses accounting for over 50% of the injuries were either a contusion (35.7%) or strain/sprain (28.0%). From here forward, the tables only give the estimated value (N); the interested reader can find the n, N as well as the 95% confidence intervals of N in the Supplemental Files.

**Figure 2:**
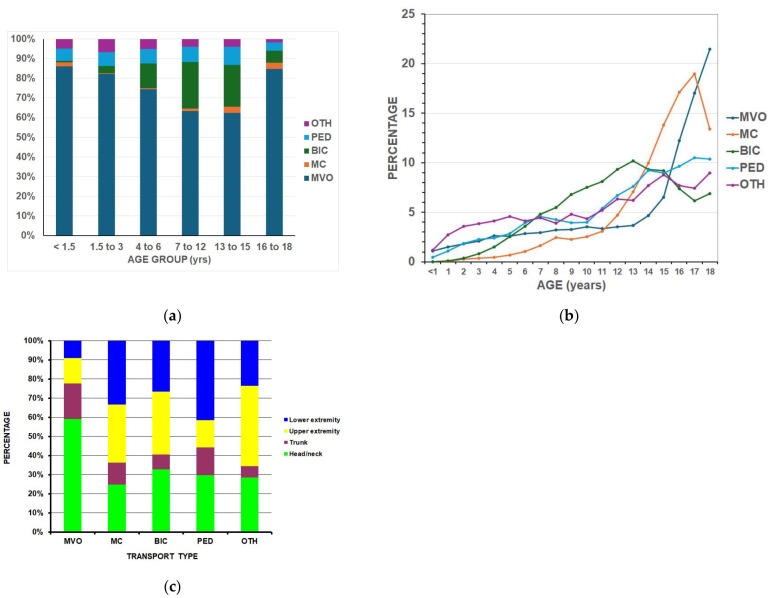
Differences between the major transportation groups: (**a**) by age group (*p* < 0.0001). MVO = motor vehicle occupant, MC = motorcycle, BIC = bicycle, PED = pedestrian, OTH = other transport; (**b**) by percentage for each year of age (*p* < 0.0001); (**c**) by anatomic area of injury (*p* < 0.0001).

Analyses between the five different transportation groups (Table 2) were performed first, as that was a major focus of the study. Using the total number of estimated ED visits for injury over this time span of 501,289,192, then the number of visits per 100,000 ED injury visits was 1221.98, 215.72, 104.04, and 39.68 per 100,000 for motor vehicle occupants, bicyclists, pedestrians, and motorcyclists respectively. Bicyclists were the youngest (12.1 years) and motorcyclists the oldest (15.7 years). The most common transportation group with injuries was a motor vehicle occupant. There was a gradual decline in the percentage of motor vehicle occupants from those <1.5 years of age to those 13–15 years old but then an increase in those 16–18 years old (Figure 2a,b). Males predominated in all groups (53.8% to 82.4%) except for motor vehicle occupants, where there was slight female predominance (55.9%). The percentage of not being discharged from the ED was highest for motorcyclists (13.8%). The head/neck was the most common area of injury in the motor vehicle group (59.2%), while the lower extremity was most common in pedestrians (41.5%) (Figure 2c).

### 3.1. Major Diagnoses

**Figure 3:**
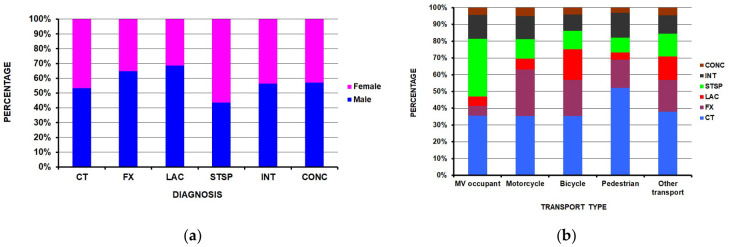
Differences between the main diagnoses: (**a**) by sex (*p* < 0.0001); (**b**) by transportation type (*p* < 0.0001).

Six major diagnoses (Table 3) accounted for 87.3% of all the injuries which were analyzed next. These were contusion (35.7%), strain/sprain (28.0%), internal organ injuries (13.3%), fracture (8.4%), lacerations (7.4%) and concussions (4.1%). All injury diagnoses were more common in males except for strain/sprains, which were more common in females (Figure 3a) (*p* < 0.0001). Within each transportation type, bicyclists had the highest proportion of lacerations, motor vehicle occupants the highest proportion of strain/sprains, and motorcyclists the highest proportion of fractures (Figure 3b) (*p* < 0.0001).

### 3.2. ED Disposition

**Figure 4:**
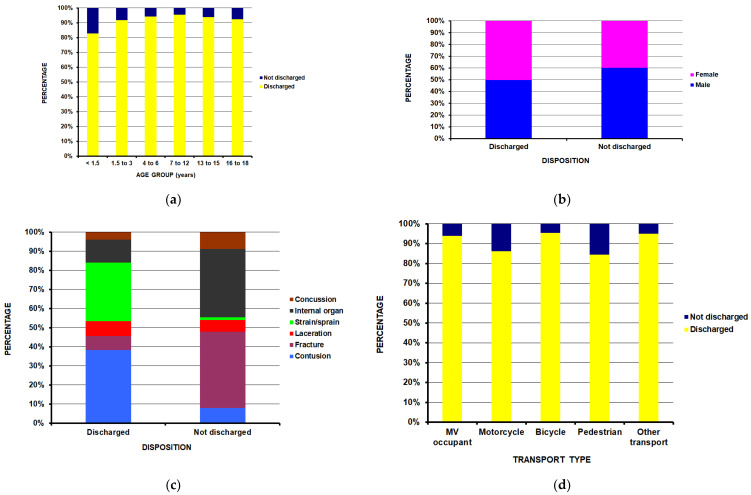
Differences by ED disposition: (**a**) by age group (*p* = 0.002); (**b**) by sex (*p* < 0.0001); (**c**) by diagnosis (*p* < 0.0001); (**d**) by transportation type (*p* < 0.0001).

Injury severity was analyzed next. Not being discharged from the ED was used as a proxy for injury severity, as those not discharged from the ED likely had more severe injuries. This proxy was necessary because injury severity scores [10,11,12,13,14] are not included in the NEISS data set. This definition has been used by other investigators with the NEISS database [15]. Not being discharged from the ED (Table 4) was most common in the youngest age group (Figure 4a) (*p* = 0.0002) and in males (Figure 4b) (*p* < 0.0001). Patients with fractures and internal organ injuries were more likely to not be discharged (Figure 4c) (*p* < 0.0001). Pedestrians were the most likely transportation group to not be discharged (Figure 4d) (*p* < 0.0001).

As not being discharged from the ED likely indicates a more severe injury, we next analyzed for predictor variables of not being discharged. As all the variables from Table 4 were significant, all were analyzed with a univariable logistic regression (Table 5). The highest predictors were the presence of a fracture (OR = 119.7 [71.3, 200.7], *p* < 0.0001), anatomic area of the trunk (OR = 3.2 [2.7, 5.8], *p* < 0.0001), transportation method—a pedestrian (OR = 3.9 [2.8, 5.3], *p* < 0.0001), those <1.5 years old (OR = 4.3 [2.8, 6.6], *p* < 0.0001), and males (OR 1.5 [1.4, 1.6], *p* < 0.0001).

Further analyses were performed to determine predictors of a fracture from the bivariate analyses (Table 6). Those most likely to sustain a fracture (Table 7) were motorcyclists (OR 5.2 [4.2, 6.4], *p* < 0.0001), males (OR 1.7 [1.6, 1.9], *p* < 0.0001), and those with an upper extremity injury (OR 11.7 [9.5, 14.4], *p* < 0.0001).

**Figure 5:**
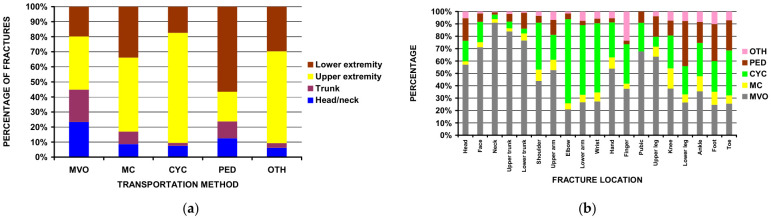
Fracture location by type of transport: (**a**) grouped anatomic location (*p* < 0.0001); (**b**) detailed location (*p* < 0.0001).

Analyses of fracture patterns (Table 8) revealed trends by transportation group. Head/neck fractures were most prevalent in motor vehicle occupants (23.3% of all fractures in motor vehicle occupants), upper extremity fractures were most prevalent in bicyclists and motorcyclists (73.1% of fractures and 49.2% of fractures, respectively), and lower extremity fractures were most prevalent in pedestrians (56.6% of all fractures in pedestrians) (*p* < 0.0001) (Figure 5a). The most common fracture in motor vehicle occupants was the face (14.7%), in motorcyclists the shoulder (13.8%), in bicyclists the wrist (21.0%), in pedestrians the tibia/fibula (29.2%), and for other transport methods the finger (20.4%) (*p* < 0.0001) (Figure 5b). While fractures of the neck accounted for only 2.0% of all fractures (13,688 of 679,412), 95.5% (12,450) of these occurred in motor vehicle occupants. Bicyclists comprised most of the individuals with fractures of the elbow, radius/ulna, and wrist (68.0%, 56.3%, and 55.9% respectively) (Figure 5b).

### 3.3. Internal Organ Injuries

**Figure 6:**
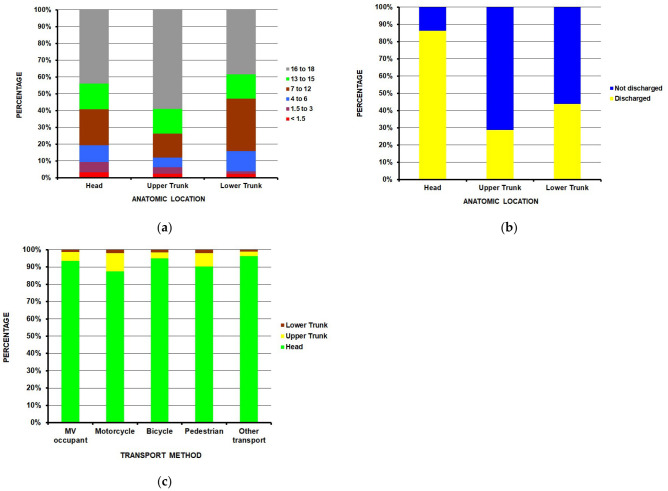
Anatomic distribution of internal organ injuries: (**a**) by age group (*p* < 0.0001); (**b**) by ED disposition (*p* < 0.0001); (**c**) by transportation method (*p* < 0.0001).

Internal organ injuries were the second-highest diagnostic predictor of not being discharged from the ED, with an OR of 65.4 [37.0, 115.9]. Of the 978,226 estimated internal organ injuries (Table 9), 913,981 (93.4%) involved the head, 50.546 (5.2%) the upper trunk, and 13,286 (1.4%) the lower trunk, with the remaining 414 involving the ear or neck. Upper trunk injuries were proportionally more common in the 16–18-year-old group (58.9% of all upper trunk injuries), while lower trunk injuries were most common in the 7–12 (31.3%) and 16–18-year age groups (38.3%) (Figure 6a) (*p* < 0.0001). Not being discharged from the ED was proportionally highest for upper trunk internal organ injuries, followed by the lower trunk and head injuries (71.3%, 56.2%, and 17.2% respectively) (Figure 6b) (*p* < 0.0001). Motorcyclists proportionately sustained more upper trunk injuries (Figure 6c) (*p* < 0.0001).

### 3.4. Hospital Strata

**Figure 7:**
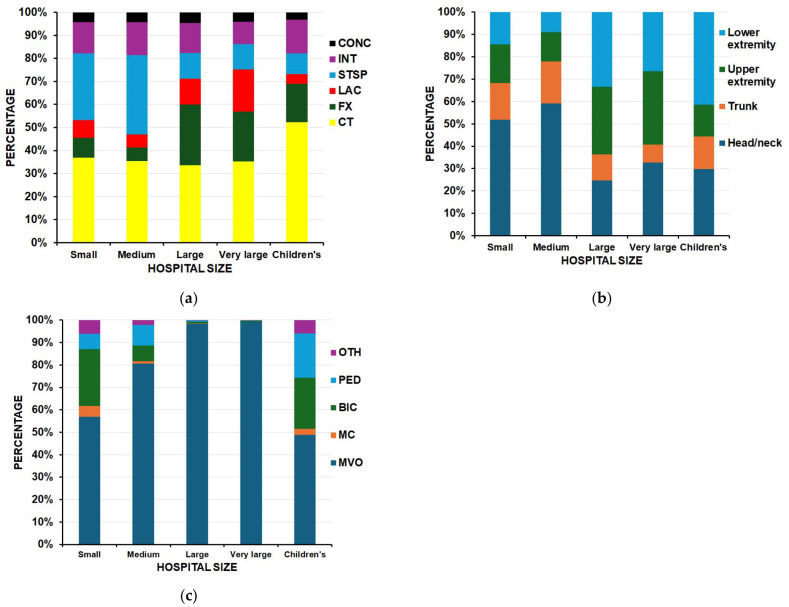
Differences by hospital size. CONC = concussion, INT = internal organ injury, STSTP = strain/sprain, LAC = laceration, FX = fracture, CT = contusion/abrasion, MVO = motor vehicle occupant, MC = motorcycle, BIC = bicycle, PED = pedestrian, OTH = other transport mechanism: (**a**) by diagnosis (*p* < 0.0001); (**b**) by anatomic location of injury (*p* < 0.0001); (**c**) by transportation method (*p* = 0.0001).

Hospital size can be used as a proxy for rural/urban locations, excluding the children’s hospital strata. Fractures were more common in the larger and children’s hospitals (*p* < 0.0001) (Table 10, Figure 7a) and the head/neck was a more common site of injury in the smaller hospitals (*p* < 0.0001) (Figure 7b). While motor vehicle occupants were the major transportation type seen across all five hospital strata (Figure 7c), children’s and small hospitals saw a greater proportion of injuries in the pedestrian, bicycle, and motorcycle groups (*p* = 0.0001). 

## 4. Discussion

Our results are discussed using a framework of highlighting other pertinent studies from the literature. These global studies allow for comparisons between the US and other countries, noting both similarities and differences. As the literature regarding injury is vast, not all studies can be cited.

### 4.1. Types of Transportation

In this study, 74.8% of the patients were motor vehicle occupants, with bicyclists, pedestrians and motorcyclists at 13.2%, 6.4% and 2.4% respectively. These percentages clearly vary depending upon the geographic outlay of a country and its cultural practices. In Singapore, among children, it was noted that 60.4% were motor vehicle occupants, 28.5% pedestrians, 9.9% cyclists, and 1.2% motorcyclists [16]. The higher percentage of pedestrians in the Singapore study is likely due to the high population density in Singapore, while the US has many less densely populated areas, such that motor vehicles are typically used for transportation.

In Iranian children <19 years of age sustaining transport-related injuries admitted to trauma hospitals [17], 54.2% were pedestrians, 24.9% were motorcyclists, 11.1% were bicyclists, and 9.9% were motor vehicle occupants. In Qatari children [18], a study of road traffic injuries at a trauma center found that 54% were due to motor vehicle crashes, 25% were pedestrians, 5% were bicyclists, 2% were motorcyclists, and 14% were ATV users. In this study, we did not review ATV injuries, and when excluding those from the Qatari study, the distribution would become 63% motor vehicle, 29% pedestrian, 6% bicyclist, and 2% motorcyclist. Our study has similar proportions for the motor vehicle (74.8%) and motorcyclists (2.4%), but different for the bicyclists (13.2%) and pedestrians (6.4%). This is likely due to different cultural preferences/needs between Qatar and the US, as well as the fact that our study encompasses all patients, not just those seen at a trauma center.

In urban São Paulo, [19] transportation-related accidents in patients ≤19 years of age, it was found that 64.4% were male. Cars and motorcycles were most frequently involved (42.8% were cars, 40.1% motorcycles). Among the victims of accidents due to cars, 55.8% were pedestrians and 44.2% were occupants. Among the accidents due to motorcycles, 52.5% related to pedestrians were run over by the motorcycle. The higher pedestrian percentage in São Paulo likely represents similar demographics as those seen in Singapore, with both being densely populated urban areas.

Motorcycle-associated injuries are often more frequent in rural areas. In rural Hunan province, China, 80% of road traffic injuries in children involved motorcycles [20]. In our study, we also noted motorcycle injuries were most commonly seen in the EDs of small hospitals. Differences in rural vs. urban locations for injuries due to motorcycles vary between countries, likely depending upon population densities, geography, and cultural practices.

### 4.2. Anatomic Location of Injury

The head/neck was the most injured location in the motor vehicle occupant group (59.2%), while the extremities were most commonly injured in the motorcycle (63.6%), bicycle (59.4%), and pedestrian (65.4%) groups. This is consistent with the study of Mitchell et al. [21] from New South Wales, Australia. Head/neck injuries were the most common moderate and severe injuries in motor vehicle occupants, followed by injuries to the abdomen. However, our results are different from those of Mitchell et al. [19] in that for the pedestrian and bicyclist groups, the extremities were more frequently injured in our study as opposed to the head/neck in Mitchell et al. [21]. One possible explanation is that Mitchell et al. [21] focused primarily on moderate and severe injuries, while this study encompassed all injuries. In a study from Sheffield Children’s Hospital, England, [22] pediatric road injures in bicyclists and pedestrians were analyzed. In the bicycle group, 22% had fractures and 19% were admitted to the hospital; in the pedestrian group, 25.6% had fractures and 45.6% were admitted. Injuries above the neck occurred in 67.4% and 49.3% of the pedestrian and bicycle groups respectively. In an Iranian study of transportation-related injuries [17], the most common injury location was the lower extremity (69.9%) in motorcyclists and pedestrians (57.1%), the head (56%) in motor vehicle occupants, and the upper extremity (41.2%) in bicyclists. This order is similar to that seen in our study.

### 4.3. Differences by Sex

In this study, motor vehicle occupants were more commonly female (55.9%), compared to the male predominance in motorcyclists (82.4%), bicyclists (75.7%), and pedestrians (60.0%). This is different from much of the literature. The male percentage of children involved in motor vehicle crashes was 49.8% in New York City children [23] and 49.8% in New Jersey [24]. For the entire US, studies have noted male percentages of 51.0% [25], 50.5% [26], and 49.3% in 75,999 children, and 49.3% of 75,999 [27], and 50.4% in a study from 11 states. In South Korea, the male percentage was 52.0 [28]. In São Paulo, Brazil [19], 64.4% were male, with transport accidents predominating among males regardless of age. In Iran [17], the male percentage was 54.8% for motor vehicle occupants. We have no explanation as to why there were more females than males in the motor vehicle occupant group in this study.

There are few studies that give male percentages specifically for child pedestrians. Males accounted for 73% of pedestrians in Iranian children [17], 56.6% of Arab Israeli children [29], 60.5% of Singapore children [30], 54.1% of Canadian children [31], 68% of Japanese children [32], 52% of Iranian children [33], 65% of Nigerian children [34], and equal male/female proportions in another Nigerian study [35]. One US study of 73,769 pedestrian injuries in those ≤19 years of age, 51% were male [36]. The 60% prevalence reported in the present study is slightly higher or similar to those of these other reports.

For bicycle injuries, a French study [37] noted that 68.4% of injured children were male. In a Norwegian study of bicyclists of all ages [38], 73% were male. These values are similar to this study’s 75.7%. In an Israeli study of only children hospitalized for bicycle-related injuries [39], 87.0% were male.

### 4.4. Differences by Age

In this study, the greatest proportion of injuries was in the 16–18-year-old group for all transportation groups. However, bicyclists and pedestrians had a greater proportion of those 7–15 years old. The same trend was seen in Qatar [18], where pedestrian-related injuries accounted for ~42% of those in the 5 to 9-year-old group and 58% in the 1–6-year-old-age group but only ~8% in the 15–18-year-old age group (percentages given are approximations, as they were interpreted from a figure in the manuscript). In our study, there was a gradual decline in the percentage of motor vehicle occupants from those <1.5 years of age to those 13–15 years old, and then an increase in those 16–18 years old (Figure 2c). This likely reflects the age at which drivers’ licenses become available to teenagers in the US. In the study from Sheffield Children’s Hospital, England [22], the median age was 7.6 years and 10.0 years for the pedestrians and bicyclists respectively, compared to the 12.8 years and 11.9 years for pedestrians and bicyclists in this study.

### 4.5. Specific Transportation Types

#### 4.5.1. Motor Vehicle Occupant Injuries

In this study, the head/neck was the most commonly injured (59.2%) anatomic site, followed by the trunk (18.6%) and extremities (27.9–18.3% upper and 8.9% lower extremity). A similar study from Korea [28] used their Emergency Department-based Injury In-depth Surveillance (EDIIS) database (similar to the NEISS data). They studied children ≤12 years of age involved in road traffic injuries involving both passenger vehicles and school buses. Head/neck injuries occurred in 73.3%, greater than the 59.2% in this study, with extremity injuries (14.4% overall; 6.5% upper extremity and 7.9% lower extremity) being lower. Hospital admission rate was 7.1%, very similar to our 6.0%. It is possible that the head/neck injury rate in the Korean study was higher due to the age criteria, being ≤12 years in the Korean study and ≤18 years in this study. However, the study of Faul et al. [40] noted that TBIs among pediatric motor vehicle occupants changed by age, with incidence per 100,000 population being 47.4 for those 0–4 years old, 7.2 for those 5–9 years old, 11.7 for those 10–14 years old, and 90.7 for those 15–19 years old, contradicting that possibility. In a study using the US National Automotive Sampling System Crash Worthiness Data System from 1997 through 2010, there were 459,973 crashes involving children 0 to 14 years of age. A head injury was defined as involving some element of brain injury or skull fracture that occurred in 15,386 (3.4%). This 3.4% is low compared to our value of 59.2%, as it is likely that many of the children were not actually injured and not taken to the hospital for evaluation, nor does it include neck and face injuries.

Faul et al. [40], as noted above, found that 29.1% of those with TBIs were hospitalized. The overall hospitalization rate in this study was 6.0% for motor vehicle occupants (Table 4) and 13.4% for concussions from all transportation injuries (Table 3). However, the admission rate for internal organ injuries was 35.9% overall, and over 90% of the internal organ injuries involved the head in the motor vehicle group (Figure 7c).

#### 4.5.2. Pedestrian Injuries

In a US study of ~301 million ED visits across all ages [41], a pedestrian-related motor vehicle injury accounted for 45.62 per 100,000 total ED visits, less than the 104.04 noted in our study. Barry et al. [41] used the National Syndromic Surveillance Program data, which receives electronic health record data from approximately 78% of EDs nationwide. However, it included patients of all ages, not just those ≤18 years old; when adjusting for those 0 to 14 years of age, the number was 29.50 per 100,000. When analyzing our data for those 0 to 14 years old, the number is 62.47 per 100,000 ED visits (313,140 out of 501,280,192). These numbers are similar, but the differences may lie in the time span studied and differences in how the data was coded/extracted between these two studies. Also, it may depend upon the known variability in the road infrastructure and physical environment where these injuries occurred. Agran et al. [42] in a study of 345 child pedestrians from Orange County, California, struck by moving vehicles found that 81% were injured on the road and 19% in driveways/parking lots. In this study, driveways/parking lots were excluded due to the study design of the NEISS traffic-related definitions; thus, the 521,529 would be higher. Events at intersections (28% of the 345) occurred more often on streets with more than two lanes, with speed limits > 25 mph and moderate/heavy traffic compared to those midblock (53% of 345). The median age of the children was 6 years for the midblock and 10 years for the intersection groups; the overall median age in this study was 12.8 years; however, Agran et al. [42] limited their study to those <15 years of age, while ours was up to 18 years of age. In Leeds and Bradford, England [43], 64% of child pedestrian casualties occurred on low-volume roads compared to 26% on radial routes exiting Leeds and Bradford. Traffic volume data is not available in our study.

#### 4.5.3. Bicycle Injuries

In this study, the upper extremity was injured in 32.9% of the cases, the head/neck in 32.8%, the lower extremity in 26.5%, and the trunk in 7.9. A Hungarian series of pediatric bicycle injuries [44] found that a contusion was the most common injury (26.4%), with fractures accounting for only 20.0% of the injuries. There were 596 injuries involving the head (28.4%), with 17.6% being mild head injuries, 9,1% concussions, and 0.9% severe head injuries (e.g., intracranial bleeds); injuries to the trunk occurred in 10.6%. They noted that poor road quality was an important contributing factor, especially in smaller villages. In a Canadian study [45], for children hospitalized for bicycle-related injuries, the rates of head injuries were highest in rural areas and lowest in urban areas (18.49 vs. 10.93 per 100,000 bicycle-related hospitalizations). In an Israeli study of children hospitalized for bicycle-related injuries [39], the head/neck was injured in 30.1%, the torso in 21.0%, the extremities in 42.5%, and traumatic brain injuries in 32.6%. The Israeli numbers are different from ours, in that concussions and internal organ injuries accounted for only 13.5% of the injuries. This is likely due to the fact that they studied only hospitalized children seen at trauma centers, skewing the data towards more severely injured patients, while our study included all patients.

#### 4.5.4. Motorcycle Injuries

Motorcycles accounted for only 2.4% of the patients in this study but had the second-highest admission rate (13.8%). The low percentage of motorcycle patients is likely due to the fact that many motorcycle injuries in children occur in off-road locations (e.g., motocross race parks, rural areas on private property) [46,47,48,49,50,51] and were therefore not included in our study. This group was the oldest, at an average age of 15.1 years.

In a study of US pediatric motorcycle passengers [52] from the American College of Surgeons database, the average age was 12 years; in this study, it was 15 years, likely due to the fact that our study excluded off-road injuries, which would have younger children, as no license would be needed to operate the motorcycle. A driver’s license is needed to legally operate a motorcycle on a US public highway/road [46,53] but not off-road. Males comprised 57%, with the preschool–kindergarten group having an even greater male percentage (74%). The percentage of fractures was 39.6% compared to the 25.8% in this study. Authors of the above study noted that school-age children (7–17 years) had higher rates of extremity injuries, possibly due in part to size ratios as well as better awareness of self-protecting responses. In a German study of pediatric motorcycle injuries [54], injuries to the lower extremities were most common (53.1%), with upper extremity injuries at 39.6%, whereas in this study, the upper and lower extremity injury prevalence was similar (30.3% and 33.3% respectively); 84% were male, similar to the 82% in the present study.

In this study, we do not know if the patient was the driver/operator or a passenger on the motorcycle. It is clear from Figure 2b that some patients were passengers due to their very young age. In a study of Taiwanese pediatric motorcycle passengers [55], 52.8% were boys and 48.5% were of preschool age; in this study, only 1.2% were ≤4 years of age, a marked difference. In the Taiwan study [55], the extremities were most frequently injured (81.0%), followed by the face (42.3%), head (39.7%), abdomen (11.5%), and chest (5.9%). In this study, the extremities were injured in 63.3%, followed by the head/neck in 24.8% and trunk in 11.6%; these numbers are different in that we only have the most severely injured anatomic location in the NEISS data, whereas Fan et al. [55] were able to account for multiple sites of injury. In Taiwan, the majority of the motorcycles are scooters that can easily carry young children passengers in the front area. Similarly, in Malaysia, the motorcycle is a commonly used transportation mode for families in Malaysia [56]. Malaysian motorcycle crashes constitute 60% of all road trauma [57], with a substantial proportion involving those ≤16 years old; 88.9% were 10–16 years of age, 82.9% were male, 38% were passengers and 75.6% occurred on rural roads [56]. The most frequently injured body area was the head (44%). In a Laotian series of pediatric road motorcycle injuries [57], 22% were ≤9 years of age; 65% were male, 67.6% were the driver/operator and 32.4% were passengers.

#### 4.5.5. Other Transportation Types

The NEISS describes these as “railway, water, air, space, animal and animal-drawn conveyances (e.g., horseback riding), ATVs, battery-powered carts, ski lifts, and other cable cars not on rails.” This group accounted for 3.2% of all the injuries in the present study, and the NEISS data is not adequately granular to delineate which of the above transportation methods were involved. When reviewing our results, the most common area of injury was the upper extremity, which is the highest percentage of all five groups (42.0%). This likely indicates that the patients were ejected from the vehicle with the upper extremity extended in order to break the fall. It is known that injuries in children from ATVs occur in the upper extremity over 50% of the time [58,59,60]. It is unlikely that these injuries were prevalent, as such transportation is rare in the US. The same applies to horseback riding; in a study [61] of pediatric fractures due to equestrian activity, only 1.3% occurred on the road. Animal-drawn buggies are common in areas of Amish population [62,63]. In a study of the Amish population from northeast Indiana and northwest Ohio [64], 36 of 42 horse-drawn buggy crashes were with a motorized vehicle, indicating that most occurred on the road. However, the Amish number compared to the entire universe of US hospitals is likely small.

### 4.6. Injury Severity and Predictors of Severe Injury

As the NEISS does not contain injury severity scores, we used not being discharged from the ED as a proxy for a severe injury. While not as exact or detailed as an injury severity score, it does give a reasonable estimate of injury severity [15]. We found that pedestrians had the highest chances of not being discharged from the ED (OR = 3.96), with bicyclists having the lowest chances (OR = 1.0 reference). This was somewhat surprising for the bicycle group to have the lowest odds of not being discharged, as the force between a bicycle rider and another vehicle or pedestrian can be quite large. The NEISS AIP [8] defines the bicycle-associated injury as “Injury to a bicycle rider from a collision, loss of control, crash, or some other event involving a moving vehicle or pedestrian. This category includes riders of unicycles, bicycles, tricycles, and mountain bikes. This category does not include injuries unrelated to transport (moving), such as repairing a bicycle.” This indicates that the bicyclist was involved in a collision or crash involving either a motor vehicle or pedestrian. A French study [37] using the Rhône road trauma registry noted that children had the highest proportion of slight/minimal injury when colliding with a motor vehicle. The percentage of serious casualties was 4.5% among children, 10.9% among adults injured outside towns and 7.2% among those injured in towns; this agrees with the bicycle group in our study having the lowest odds of not being discharged. A New York City study [65] of child pedestrians or bicyclists struck by motor vehicles noted that injury severity scores were not different between the bicyclist and pedestrian groups, in contrast to our findings; however, the number of patients in that study was small (145 pedestrians, 17 bicyclists) and only involved a major metropolitan area with high population density.

Those with fractures had the highest risk of not being discharged (OR = 119.7), with internal organ injuries and concussions being the next highest (OR = 65.4 and 48.7 respectively). Patients with injuries to the trunk had the highest odds of not being discharged when compared to other anatomic locations (OR = 3.2), which likely reflects that fractures to the spine as well as internal organ injuries involving the trunk (e.g., spleen, liver, lung, etc.) are frequently admitted to a hospital for continuing observation/treatment. Predictors of a fracture were highest with an upper extremity injury (OR = 11.7). Motorcyclists, bicyclists, and pedestrians had similar ORs (5.2, 4.3, 3.1 respectively) of not being discharged, with motor vehicle occupants having the lowest (OR = 1.0, reference). Thus, while 74.8% of the patients in this study were motor vehicle occupants, they had the lowest chances of sustaining a fracture or not being discharged from the ED. Motor vehicle occupants having the lowest odds of not being discharged may reflect the protective nature of the automobile itself as well as safety equipment (seat belts, air bags). Pedestrians had the highest proportion of head fractures, supporting our finding that pedestrians had the highest odds of not being discharged from the hospital.

### 4.7. Prevention Strategies

While it was not the purpose of this study to delve deeply into injury prevention, a few comments should be made. As 75% of the injured patients were motor vehicle occupants, prevention strategies should be strongly directed to this group. It is clearly known that seat belt use decreases the injury rate in motor vehicle crashes, with 50% of all motor vehicle occupants killed in traffic crashes being unrestrained [66]. Despite having the highest risk per miles driven for motor vehicle crash involvement, only 57% of US high school students reported always using a seat belt when riding in a car with another driver [67]. Thus, encouragement, education, and enforcement of belt restraints is a major avenue of prevention.

For children, as they develop, belt restraints involve child safety seats, then booster seats and final progression to standard adult shoulder harness and/or lap belts. The NEISS data is not adequately granular to know the child restraint status in the motor vehicle occupant group. Injuries to the head/neck comprised 59.2% of the injuries in the motor vehicle group in this study, which agrees with many other studies. In one study of pediatric motor vehicle crash patients, up to 25.8% were reported as unrestrained [68]. In a pediatric finite element model analysis using a child restraint system [69], forward-facing models showed higher head accelerations in frontal crash simulations, suggesting that rear-facing positions should be considered at least until the age of 48 months. In another study of motor vehicle fatal side impact crashes with at least one fatally injured restrained rear seat occupant between the ages of 6 and 12 [70], 61% were in vehicles with side airbags. Perhaps having more vehicles with side airbags might lower this number, but of course, not every family is able to have a vehicle with the most up-to-date safety features due to cost and other issues. In a study of craniofacial injuries and seat belt use and seat location [71], it was noted that those in the back seat had a greater risk of a facial bone fracture than those in the passenger’s seat. While this study spanned all ages, most children up until their teen years are sitting in the back seats. This may explain the high incidence of head and neck injuries noted in our study. In a study of infants and young children in road traffic injuries [72], those using car safety seats were less likely to have intracranial injuries compared with those not using car safety seats. In a study of severely injured children who were rear seat occupants in motor vehicle collisions [73], children were more likely to have severe head injuries associated with a lack of an age-appropriate child restraint, and adolescents were more likely to have severe abdominal injuries associated with the use of lap–shoulder belts. So, clearly enforcing the use of car safety seats is preventive of head/neck injuries in children.

The association of child pedestrian injuries with low socioeconomic status has been noted in many studies. [43,74,75,76,77,78,79,80,81,82,83,84]. The same has been noted for children sustaining bicycle road traffic injuries [85]. Children from lower socioeconomic groups often live in neighborhoods with poor infrastructure, e.g., lack of barriers between pedestrian paths and the street, poor street lighting, lack of crossing signals, more road traffic, poor sidewalk infrastructure, and often non-existent sidewalks. Thus, these children are at increased risk of injury on the road. Children often dart out [86] to retrieve a toy when playing close to a road; this is more likely in younger children [36]. The amount of outdoor space available for play in a home’s yard is likely much less for children in lower socioeconomic areas, as yard lots are often smaller and the population density is increased compared to much larger homes and lots in upper socioeconomic class neighborhoods. While the NEISS data is not adequately granular to determine socioeconomic status of these children, clearly improving infrastructure in neighborhoods where needed is another method of injury prevention.

Regarding motorcycle-associated injuries, helmet use, protective clothing, training, and penalties for alcohol consumption and speeding are well known to reduce injuries [87]. Protective clothing has been shown to reduce injuries in general but not for fractures [88,89,90]. The role of back protectors may be beneficial [91,92]. As fracture was the most common diagnosis in the motorcycle group in this study, protective clothing may not have a huge impact on prevention of those injuries, but certainly could reduce contusions and lacerations.

Education is another prevention strategy for all the transportation groups [93,94,95]. Such programs could be incorporated into school curriculums. However, such education programs may need to be repeated on a yearly basis [96].

### 4.8. Limitations

Fatal injuries are not included in the NEISS AIP data. There is no denominator used in the weighted estimates, and thus, this is simply a prevalence rather than an incidence study. Granular detail for many parameters is not known, such as time of injury, weather conditions, collision details for the pedestrian and bicycle groups (i.e., a pedestrian vs. car, pedestrian vs. bicycle, motor vehicle vs. bicycle, etc.), road condition, presence/absence of stop signs/traffic signals, etc. Additionally, the NEISS only captures those patients seen in US hospitals with Eds; thus, patients seen in other centers (e.g., urgent care centers, private physician offices) will not be captured. However, it is likely that most major/serious injuries would be seen initially in an ED. While not ideal, being discharged from the ED was the measure used for injury severity. Not being discharged was defined as being admitted, observed, or transferred to another hospital for further care, all of which are of greater severity than simply being discharged from the ED. Not being discharged may depend upon the definition of admission versus observation as well as insurance issues. Thus, there is potential for misclassification using ED discharge in this fashion and thus potentially different findings. However, there is likely minimal error, as very few contusions/abrasions, strain/sprains, and lacerations were not discharged from the ED (0.35 to 5%), whereas fractures, internal organ injuries and concussions had much higher percentages of not being discharged (13.4% to 27.5%). This is what would be expected in any trauma situation and agrees with the high OR for fractures not being discharged from the ED.

## 5. Conclusions

There were an estimated 8,188,810 ED visits for traffic-related injuries in children; the median age was 14.3 years. Sex distribution was equal; 93.4% were discharged from the ED. The head/neck was the most injured area (51.9%) and the most common diagnoses were contusion (35.7%), strain/sprain (28.0%), internal organ injuries (13.3%), fracture (8.4%), lacerations (7.4%) and concussions (4.1%). Predictor variables of severe injury (not being discharged from the ED) were the presence of a fracture (OR = 119.7), trunk injury (OR = 3.2), a pedestrian (OR = 3.9), those <1.5 years old (OR = 4.3), and males (OR 1.5). The results from this study provide insights into the differences in demographic and injury profiles of road traffic injuries among US children. They can be used to compare/contrast these injuries to other countries, and also to assess the outcomes of future prevention strategies.

## Figures and Tables

**Table 1:** Overall demographics of pediatric road traffic-related injuries.

	All				
Parameter	n	N	L	U	%
**All**	167,464	8,188,810	7,584,758	8,768,157	
**Age (yrs)**					
**Average (95%CI)**	13.0 [12.8, 13.2]				
**Median**	14.3				
**Age group (yrs)**					
**<1.5**	3504	139,608	113,824	171,965	1.7
**1.5 to 3**	9121	349,981	311,994	392,244	4.3
**4 to 6**	16,288	650,640	607,610	696,868	7.9
**7 to 12**	44,832	1,919,807	1,807,270	2,032,463	23.4
**13 to 15**	29,694	1,464,124	1,402,743	1,528,032	17.9
**16 to 18**	64,025	3,664,649	3,478,606	3,852,835	44.8
**Sex**					
**Male**	85,620	4,136,884	4,026,833	4,247,089	50.5
**Female**	81,794	4,051,064	3,940,859	4,161,115	49.5
**ED Disposition**					
**Discharged**	139,102	7,024,184	6,767,991	7,197,335	93.4
**Not discharged**	15,195	494,971	321,820	751,164	6.6
**Anatomic area of injury**					
**Head/neck**	86,369	4,157,272	3,958,788	4,354,987	51.9
**Trunk**	25,805	1,308,219	1,227,016	1,393,500	16.3
**Upper extremity**	26,162	1,384,191	1,299,853	1,472,740	17.3
**Lower extremity**	24,752	1,154,339	1,028,517	1,292,649	14.4
**Diagnosis**					
**Contusion**	52,326	2,632,579	2,430,895	2,842,072	35.7
**Fracture**	15,043	618,590	596,466	778,063	8.4
**Laceration**	10,961	549,886	505,667	597,942	7.4
**Strain/sprain**	38,134	2,065,410	1,831,476	2,316,473	28.0
**Internal organ**	23,114	978,391	746,321	1,268,967	13.3
**Concussion**	7011	303,003	250,988	365,409	4.1
**Unknown**	4543	171,147	120,327	242,130	2.3

n = actual number of ED visits, N = estimated number of ED visits, L and U are the 95% confidence intervals of N.

**Table 2:** Results by transportation type.

	Motor Vehicle Occupant		Motorcyclist		Bicyclist		Pedestrian		Other Transport		-
Parameter	N	%	N	%	N	%	N	%	N	%	*p* Value
**All ^**	6,125,656	74.8	198,922	2.4	1,081,362	13.2	521,529	6.4	261,341	3.2	<0.0001
**Average (95%CI)**	13.3		15.1		12.1		12.1		11.0		
**Median**	15.1		15.7		11.9		12.8		11.5		
**Age group (yrs)**											
**<1.5**	120,281	2.0	2584	1.3	1211	0.1	8837	1.7	6696	8.9	<0.0001
**1.5 to 3**	287,682	4.7	1063	0.5	13,324	1.2	24,672	4.7	23,239	12.8	
**4 to 6**	484,262	7.9	3428	1.7	81,944	7.6	47,499	9.1	33,507	12.8	
**7 to 12**	1,214,580	19.8	26,157	13.1	454,075	42.0	149,145	28.6	75,850	29.0	
**13 to 15**	912,882	14.9	48,248	24.3	310,420	28.7	133,392	25.6	59,182	22.6	
**16 to 18**	3,105,970	50.7	117,441	59.0	220,387	20.4	157,983	30.3	62,867	24.1	
**Sex**											
**Male**	2,700,877	44.1	163,884	82.4	818,486	75.7	312,928	60.0	140,709	53.8	<0.0001
**Female**	3,424,041	55.9	35,028	17.6	262,866	24.3	208,496	40.0	120,632	46.2	
**ED Disposition**											
**Discharged**	5,295,720	94.0	154,708	86.2	942,136	95.5	409,227	84.5	222,394	95.0	0.0001
**Not discharged**	338,452	6.0	24,755	13.8	44,883	4.5	75,224	15.5	11,657	5.0	
**Anatomic area injured**											
**Head/neck**	3,531,275	59.2	48,452	24.8	350,875	32.8	152,758	29.8	73,911	28.5	<0.0001
**Trunk**	1,110,838	18.6	22,672	11.6	84,523	7.9	74,449	14.5	15,737	6.1	
**Upper extremity**	791,353	13.3	59,239	30.3	351,819	32.9	72,749	14.2	109,030	42.0	
**Lower extremity**	532,588	8.9	65,154	33.3	283,436	26.5	212,511	41.5	60,624	23.4	
**Diagnosis**											
**Contusion**	1,926,299	34.6	56,450	33.0	333,354	34.6	236,557	50.4	79,919	36.9	<0.0001
**Fracture**	317,685	5.7	44,200	25.8	204,018	21.2	75,810	16.1	39,878	18.4	
**Laceration**	308,304	5.5	19,026	11.1	174,187	18.1	19,002	4.0	29,357	13.5	
**Strain/sprain**	1,873,194	33.7	18,565	10.8	104,213	10.8	40,888	8.7	28,551	13.2	
**Internal organ**	776,055	14.0	21,812	12.7	91,106	9.5	66,439	14.2	22,977	10.6	
**Concussion**	232,330	4.2	7975	4.7	38,849	4.0	14,143	3.0	9705	4.5	
**Unknown**	126,993	2.3	3138	1.8	17,953	1.9	16,595	3.5	6468	3.0	

^ percentages are row percentage for all; the remainder are column percentages.

**Table 3:** Results by diagnosis.

Parameter	N	%	N	%	N	%	N	%	N	%	N	%	*p* Value
_	Contusion		Fracture		Laceration		Strain/Sprain		Internal Organ		Concussion		
**Age (yrs)**													
**Average**	12.2		13.2		12.2		14.3		12.5		14.5		<0.0001
**Median**	13.1		13.9		13.0		15.4		14.2		15.4		
**Age group (yrs)**													
**<1.5**	60,054	2.3	9440	1.4	6930	1.3	5011	0.2	30,277	3.1	2173	1.9	<0.0001
**1.5 to 3**	161,093	6.1	19,203	2.8	28,073	5.1	27,200	1.3	58,949	6.0	5680	4.1	
**4 to 6**	257,646	9.8	39,354	5.8	63,916	11.6	93,721	4.5	96,831	9.9	12,496	4.1	
**7 to 12**	686,931	26.1	183,987	27.0	148,154	26.9	426,980	20.7	206,014	21.1	48,770	16.1	
**13 to 15**	452,885	17.2	155,103	22.8	93,403	17.0	385,800	18.7	150,288	15.4	59,663	19.7	
**16 to 18**	1,013,970	38.5	274,505	40.3	209,410	38.1	1,126,698	54.6	463,033	47.3	174,221	57.5	
**Sex**													
**Male**	2,160,890	53.4	1,120,135	64.9	1,018,894	68.6	1,142,497	43.5	797,971	56.5	270,013	57.1	<0.0001
**Female**	1,888,119	46.6	605,176	35.1	465,866	31.4	1,481,070	56.5	614,072	43.5	202,462	42.9	
**ED Disposition**													
**Discharged**	2,579,373	98.6	493,306	72.5	518,290	94.8	2,052,243	99.7	804,246	82.8	262,026	86.6	<0.0001
**Not discharged**	36,579	1.4	187,147	27.5	28,314	5.2	6507	0.3	166,846	17.2	40,433	13.4	
**Anatomic area**													
**Head/neck**	846,161	33.4	105,346	15.5	354,922	64.7	1,199,317	58.9	914,394	93.5	303,003	100.0	<0.0001
**Trunk**	574,237	22.6	85,567	12.6	728	0.1	430,434	21.1	63,832	6.5	0	0.0	* <0.0001
**Upper extremity**	560,500	22.1	322,402	47.3	85,328	15.5	227,398	11.2	0	0.0	0	0.0	
**Lower extremity**	555,388	21.9	167,987	24.7	100,789	18.4	178,485	8.8	0	0.0	0	0.0	
**Mechanism**													
**MV occupant**	1,926,299	73.2	317,685	46.6	308,304	56.1	1,873,194	90.7	776,055	79.3	232,330	76.7	<0.0001
**Motorcycle**	56,450	2.1	44,200	6.5	10,296	1.9	18,566	0.9	21,812	2.2	7975	2.6	
**Bicycle**	333,354	12.7	204,018	29.9	174,187	31.7	104,212	5.0	91,106	9.3	38,849	12.8	
**Pedestrian**	236,557	9.0	75,810	11.1	19,012	3.5	40,888	2.0	66,440	6.8	14,143	4.7	
**Other transport**	79,919	3.0	39,878	5.9	29,358	5.3	28,551	1.4	22,977	2.3	9705	3.2	

* excluding concussions.

**Table 4:** Results by ED disposition.

	Discharged		Not Discharged		*p* Value
Variable	N	%	N	%	
**Age (years)**					
**Average**	13.0		13.1		0.76
**Median**	14.2		15.0		
**Age group (yrs)**					
**<1.5**	106,217	1.5	21,949	4.4	0.002
**1.5 to 3**	296,775	4.2	26,172	5.3	
**4 to 6**	566,771	8.1	34,620	7.0	
**7 to 12**	1,698,830	24.2	80,947	16.4	
**13 to 15**	1,264,918	18.0	82,331	16.6	
**16 to 18**	3,090,674	44.0	248,953	50.3	
**Sex**					
**Male**	3,496,113	49.8	297,106	60.1	<0.0001
**Female**	3,527,644	50.2	197,597	39.9	
**Anatomic area of injury**					
**Head/neck**	3,573,919	52.0	251,051	52.0	0.0004
**Trunk**	1,090,356	15.9	112,942	23.4	
**Upper extremity**	1,224,108	17.8	39,911	8.3	
**Lower extremity**	979,150	14.3	78,860	16.3	
**Diagnosis**					
**Contusion**	2,579,373	38.4	36,579	7.9	<0.0001
**Fracture**	493,306	7.4	187,147	40.2	
**Laceration**	518,290	7.7	28,314	6.1	
**Strain/sprain**	2,052,243	30.6	6507	1.4	
**Internal organ**	804,246	12.0	166,846	35.9	
**Concussion**	262,026	3.9	40,433	8.7	
**Mechanism**					
**MV occupant**	5,295,720	75.4	338,452	68.4	0.0001
**Motorcycle**	154,708	2.2	24,755	5.0	
**Bicycle**	942,136	13.4	44,883	9.1	
**Pedestrian**	409,227	5.8	75,224	15.2	
**Other transport**	222,394	3.2	11,657	2.4	

**Table 5:** Logistic regression analyses for predictors of not being discharged from the ED.

Variable	OR	CI	*p* Value
**Transportation method**			<0.0001
**MV occupant**	1.3	[1.0, 1.89]	0.046
**Motorcycle**	3.4	[2.6, 4.3]	<0.0001
**Bicycle**	1.00	R	-
**Pedestrian**	3.9	[2.8, 5.3]	<0.0001
**Other transport**	1.1	[0.82, 1.5]	0.51
**Sex**			<0.0001
**Male**	1.5	[1.4, 1.36]	<0.0001
**Female**	1.00	R	-
**Diagnosis**			<0.0001
**Contusion**	4.52	[3.3, 6.0]	<0.0001
**Fracture**	119.7	[71.3, 200.7]	<0.0001
**Laceration**	17.2	[11.7, 25.4]	<0.0001
**Strain/sprain**	1.00	R	-
**Internal organ**	65.4	[37.0, 115.9]	<0.0001
**Concussion**	48.7	[34.0, 69.7]	<0.0001
**Anatomic area of injury**			<0.0001
**Head/neck**	2.2	[1.9, 2.5]	<0.0001
**Trunk**	3.2	[2/7, 3.8]	<0.0001
**Upper extremity**	1.00	R	-
**Lower extremity**	2.5	[2.1, 2.9]	<0.0001
**Age group (yrs)**			<0.0001
**<1.5**	4.3	[2.8, 6.6]	<0.0001
**1.5 to 3**	1.9	[1.5, 2.3]	<0.0001
**4 to 6**	1.3	[1.0, 1.4]	0.0001
**7 to 12**	1.00	R	-
**13 to 15**	1.4	[1.2, 1.5]	0.0001
**16 to 18**	1.7	[1.3, 2.1]	0.00001

OR = odds ratio, CI = 95% confidence interval of the OR, R = reference value.

**Table 6:** Results between those with or without a fracture.

	Fracture Diagnosis					All Other Diagnoses					
Parameter	n	N	L	U	%	n	N	L	U	%	*p* Value
**Age (yrs)**	13.2 [12.8, 13.4]					13.0 [12.8, 13.2]					0.26
**Age group (yrs)**											
**<1.5**	284	9440	5725	15,472	1.4	3220	130,169	106,603	159,153	1.7	<0.0001
**1.5 to 3**	631	19,203	15,336	23,992	2.8	8490	330,778	295,034	370,106	4.4	
**4 to 6**	1157	39,354	33,125	46,689	5.8	15,131	611,287	570,549	654,630	8.1	
**7 to 12**	4371	183,987	161,128	208,771	27.0	40,461	1,735,821	1,638,075	1,837,767	23.1	
**13 to 15**	3260	155,103	142,180	168,830	22.8	26,434	1,309,021	1,255,958	1,364,062	17.4	
**16 to 18**	5340	274,505	244,077	306,034	40.3	58,685	3,390,145	3,219,096	3,562,176	45.2	
**Sex**											
**Male**	9323	426,876	413,037	440,432	62.6	76,297	3,710,008	3,609,867	3,810,290	49.4	<0.0001
**Female**	5716	254,591	241,035	268,430	37.4	76,078	3,796,473	3,696,192	3,896,615	50.6	
**Anatomic area**											
**Head/neck**	2554	105,346	91,090	121,476	15.5	83,815	4,051,926	3,845,892	4,255,964	55.3	<0.0001
**Trunk**	1896	85,567	67,313	107,850	12.6	23,909	1,222,652	1,136,486	1,314,428	16.7	
**Upper extremity**	6181	322,402	282,808	362,452	47.3	19,945	1,061,789	996,622	1,129,896	14.5	
**Lower extremity**	4407	167,987	149,546	187,903	24.7	20,345	986,352	866,278	1,120,376	13.5	
**Mechanism**											
**MV occupant**	7205	317,685	258,254	378,555	46.6	116,126	5,807,972	5,495,285	6,084,602	77.4	<0.0001
**Motorcycle**	816	44,200	38,237	51,051	6.5	2570	154,722	135,881	176,420	2.1	
**Bicycle**	3685	204,018	153,153	263,503	29.9	15,931	877,344	706,429	1,083,292	11.7	
**Pedestrian**	2510	75,810	51,187	110,213	11.1	12,961	445,719	291,280	1,594,534	5.9	
**Other transport**	827	39,878	32,444	48,870	5.9	4833	221,464	184,678	265,756	3.0	

**Table 7:** Logistic regression analyses for predictors of a fracture.

Variable	OR	CI	*p* Value
**Transportation method**			<0.0001
**MV occupant**	1.00	R	-
**Motorcycle**	5.2	[4.2, 6.4]	<0.0001
**Bicycle**	4.3	[3.3, 5.6]	<0.0001
**Pedestrian**	3.1	[2.6, 3.7]	<0.0001
**Other transport**	3.3	[2.6, 4.2]	<0.0001
**Age group (yrs)**			0.014
**<1.5**	1.3	[0.8, 1.9]	0.30
**1.5 to 3**	1.0	R	-
**4 to 6**	1.1	[0.9, 1.4]	0.38
**7 to 12**	1.8	[1.4, 2.3]	<0.0001
**13 to 15**	2.0	[1.7, 2.5]	<0.0001
**16 to 18**	1.4	[1.3, 1.6]	<0.0001
**Sex**			<0.0001
**Male**	1.7	[1.6, 1.9]	<0.0001
**Female**	1.00	R	-
**Anatomic area of injury**			<0.0001
**Head/neck**	1.00	R	-
**Trunk**	2.7	[2.1, 3.5]	<0.0001
**Upper extremity**	11.7	[9.5, 14.4]	<0.0001
**Lower extremity**	6.6	[5.6, 7.6]	<0.0001

OR = odds ratio, CI = 95% confidence interval of the OR, R = reference value.

**Table 8:** Fracture location by type of transport.

	Motor Vehicle Occupant		Motorcyclist		Bicyclist		Pedestrian		Other Transport		*p* Value
	N	%	N	%	N	%	N	%	N	%	
**Anatomic location**											
**Head/neck**	74,017	23.3	3793	8.6	15,261	7.5	9442	12.5	2472	6.2	<0.0001
**Trunk**	68,173	21.5	3695	8.4	3931	1.9	8560	11.3	1208	3.0	
**Upper extremity**	112,348	35.4	21,748	49.2	149,060	73.1	14,896	19.7	24,349	61.1	
**Lower extremity**	62,880	19.8	14,963	33.9	35,406	17.4	42,889	56.6	11,848	29.7	

**Table 9:** Internal organ injuries.

	All		Head		Upper Trunk		Lower Trunk		
Parameter	N	%	N	%	N	%	N	%	*p* Value
**Sex**									
**Male**	515,169	52.7	477,121	52.2	30,064	59.5	7624	57.4	0.0006
**Female**	462,891	47.3	436,708	47.8	20,467	40.5	5662	42.6	
**Mechanism**									
**MV occupant**	775,897	79.3	726,233	79.5	39,485	78.1	9875	74.3	<0.0001
**Motorcycle**	21,812	2.2	19,083	2.1	2275	4.5	433	3.3	
**Bicycle**	91,106	9.3	86,551	9.5	3068	6.1	1399	10.5	
**Pedestrian**	66,433	6.8	59,982	6.6	5134	10.2	1318	9.9	
**Other transport**	22,977	2.3	22,131	2.4	585	1.2	261	2.0	
**ED Disposition**									
**Discharged**	804,088	82.8	783,601	86.4	14,452	28.7	5816	43.8	<0.0001
**Not discharged**	166,840	17.2	123,333	13.6	35,873	71.3	7449	56.2	
**Age group (yrs)**									
**<1.5**	30,277	3.1	28,767	3.1	1212	2.4	286	2.2	<0.0001
**1.5 to 3**	58,949	6.0	56,713	6.2	1982	3.9	223	1.7	
**4 to 6**	96,794	9.9	92,258	10.1	2842	5.6	1598	12.0	
**7 to 12**	205,999	21.1	194,537	21.3	7244	14.3	4164	31.3	
**13 to 15**	150,288	15.4	140,834	15.4	7475	14.8	1929	14.5	
**16 to 18**	436,009	44.6	400,872	43.9	29,786	58.9	5086	38.3	

**Table 10:** Results by hospital size.

	Small		Medium		Large		Very large		Children’s			
Parameter	N	%	N	%	N	%	N	%	N	%	*p* Value	*p* Value ^
**Age (yrs)**												
**Average [95%CI]**	13.0 [12.8, 13.2]		13.3 [13.0, 13.5]		15.1 [14.9, 15.3]		12.1 [11.7, 12.4]		12.1 [11.7, 12.5]		<0.0001	<0.0001
**Median**	14.3		15.1		15.7		11.9		12.8			
**Age group (yrs)**												
**<1.5**	139,608	1.7	120,281	2.0	2584	1.3	1211	0.1	8837	1.7	0.0004	<0.0001
**1.5 to 3**	349,981	4.3	287,682	4.7	1063	0.5	13,324	1.2	24,672	4.7		
**4 to 6**	650,640	7.9	484,262	7.9	3428	1.7	81,944	7.6	47,499	9.1		
**7 to 12**	1,919,807	23.4	1,214,580	19.8	26,157	13.1	454,075	42.0	149,145	28.6		
**13 to 15**	1,464,124	17.9	912,882	14.9	48,248	24.3	310,420	28.7	133,392	25.6		
**16 to 18**	3,664,649	44.8	3,105,970	50.7	117,441	59.0	220,387	20.4	157,983	30.3		
**Sex**												
**Male**	4,136,884	50.5	2,700,877	44.1	163,884	82.4	818,486	75.7	312,928	60.0	0.008	0.13
**Female**	4,051,064	49.5	3,424,041	55.9	35,028	17.6	262,866	24.3	208,496	40.0		
**ED Disposition**												
**Discharged**	7,024,184	93.4	5,295,720	94.0	154,708	86.2	942,136	95.5	409,227	84.5	<0.0001	<0.0001
**Not discharged**	494,971	6.6	338,452	6.0	24,755	13.8	44,883	4.5	75,224	15.5		
**Diagnosis**												
**Contusion**	2,632,579	35.7	1,926,299	34.6	56,450	33.0	333,354	34.6	236,557	50.4	<0.0001	0.016
**Fracture**	618,590	8.4	317,685	5.7	44,200	25.8	204,018	21.2	75,810	16.1		
**Laceration**	549,886	7.4	308,304	5.5	19,026	11.1	174,187	18.1	19,002	4.0		
**Strain/sprain**	2,065,410	28.0	1,873,194	33.7	18,565	10.8	104,213	10.8	40,888	8.7		
**Internal organ**	978,391	13.3	776,055	14.0	21,812	12.7	91,106	9.5	66,439	14.2		
**Concussion**	303,003	4.1	232,330	4.2	7975	4.7	38,849	4.0	14,143	3.0		
**Unknown**	171,147	2.3	126,993	2.3	3138	1.8	17,953	1.9	16,595	3.5		
**Anatomic area**												
**Head/neck**	4,157,272	51.9	3,531,275	59.2	48,452	24.8	350,875	32.8	152,758	29.8	<0.0001	0.0009
**Trunk**	1,308,219	16.3	1,110,838	18.6	22,672	11.6	84,523	7.9	74,449	14.5		
**Upper extremity**	1,384,191	17.3	791,353	13.3	59,239	30.3	351,819	32.9	72,749	14.2		
**Lower extremity**	1,154,339	14.4	532,588	8.9	65,154	33.3	283,436	26.5	212,511	41.5		
**Mechanism**												
**MV occupant**	1,373,309	56.9	1,444,701	80.5	1,873,456	98.4	1,228,495	99.1	205,698	48.8	0.0001	0.0006
**Motorcycle**	119,696	5.0	20,732	1.2	1092	0.1	975	0.1	11,550	2.7		
**Bicycle**	607,581	25.2	125,172	7.0	16,580	0.9	6126	0.5	95,881	22.8		
**Pedestrian**	167,385	6.9	163,404	9.1	9653	0.5	2289	0.2	83,099	19.7		
**Other transport**	146,694	6.1	39,965	2.2	2956	0.2	2105	0.2	25,158	6.0		

^ children’s hospitals excluded.

## Data Availability

The data is in the public domain and available at the NEISS website www.icpsr.umich.edu/web/ICPSR/series/198 (accessed on 14 May 2026).

## Supplementary Materials

The following supporting information can be downloaded at: https://www.mdpi.com/article/10.3390/children13050687/s1.
