# Supplementary material for: Demographics, Injury Patterns, Injury Severity and Injury Predictors in Children with Non-Fatal Injuries Due to Road Traffic Injuries: An Analysis by Mode of Transportation
Source: Children (Basel). 2026 May 16;13(5):687. doi: 10.3390/children13050687 (PMC13204132; doi:10.3390/children13050687)

Supplemental Figures
Comparing the traditional age group classifications to those used in this study.

Supplemental figure 1a: Percentage of ED visits by age group ($p < 0.0001$) for type of transport.

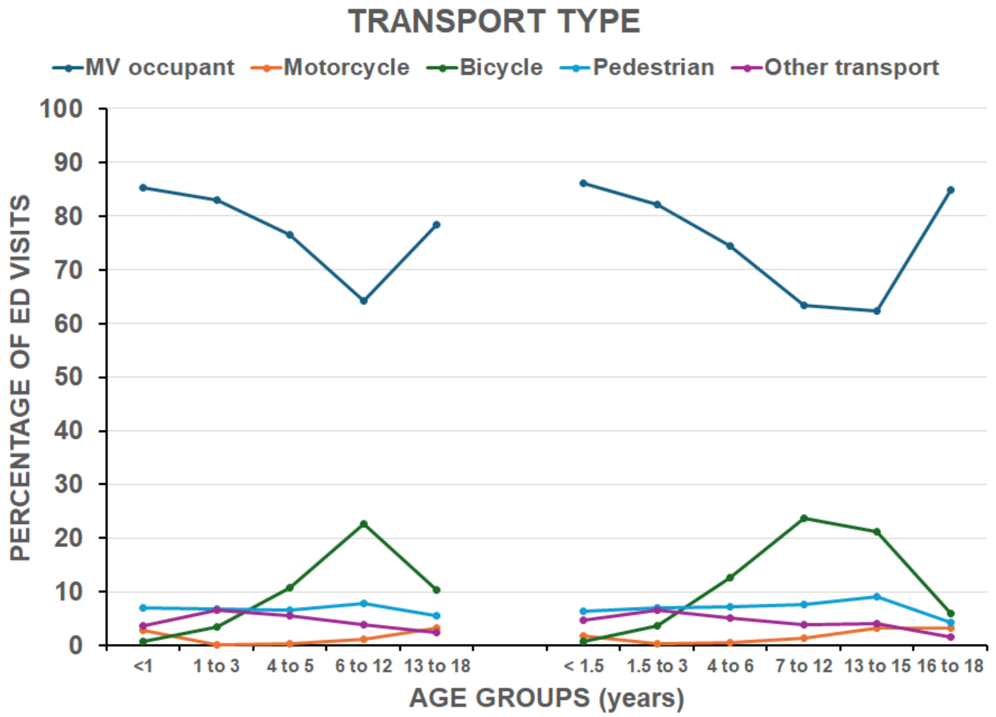

Supplemental figure 1b: Percentage of ED visits by age group ($p < 0.0001$) by sex.

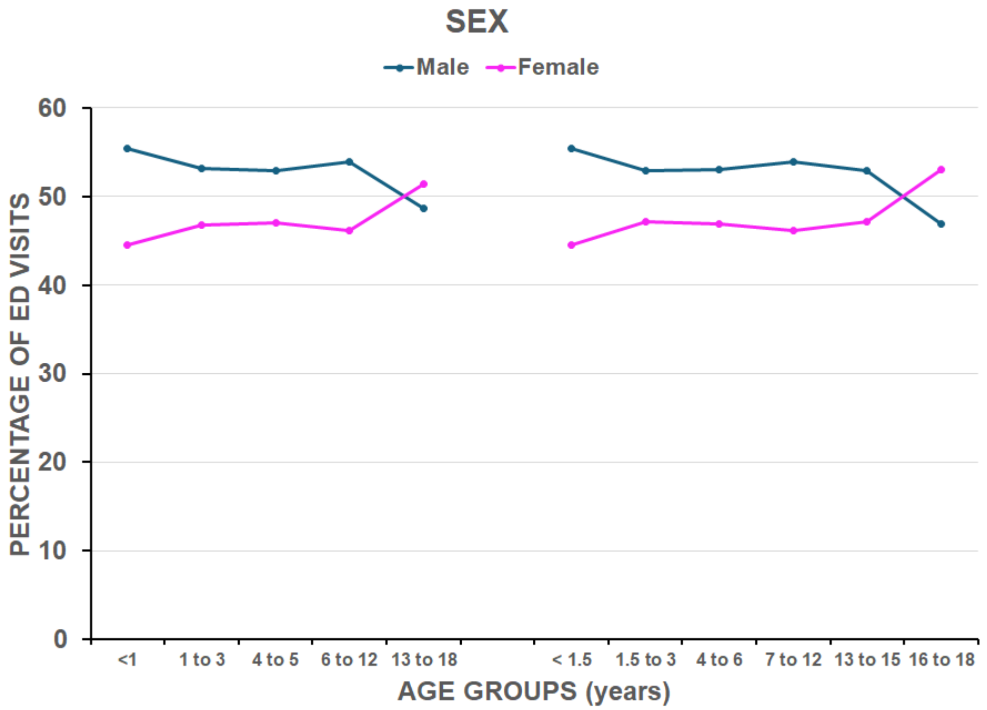

Supplemental figure 1c: Percentage of ED visits by age group ($p < 0.0001$) by diagnosis.

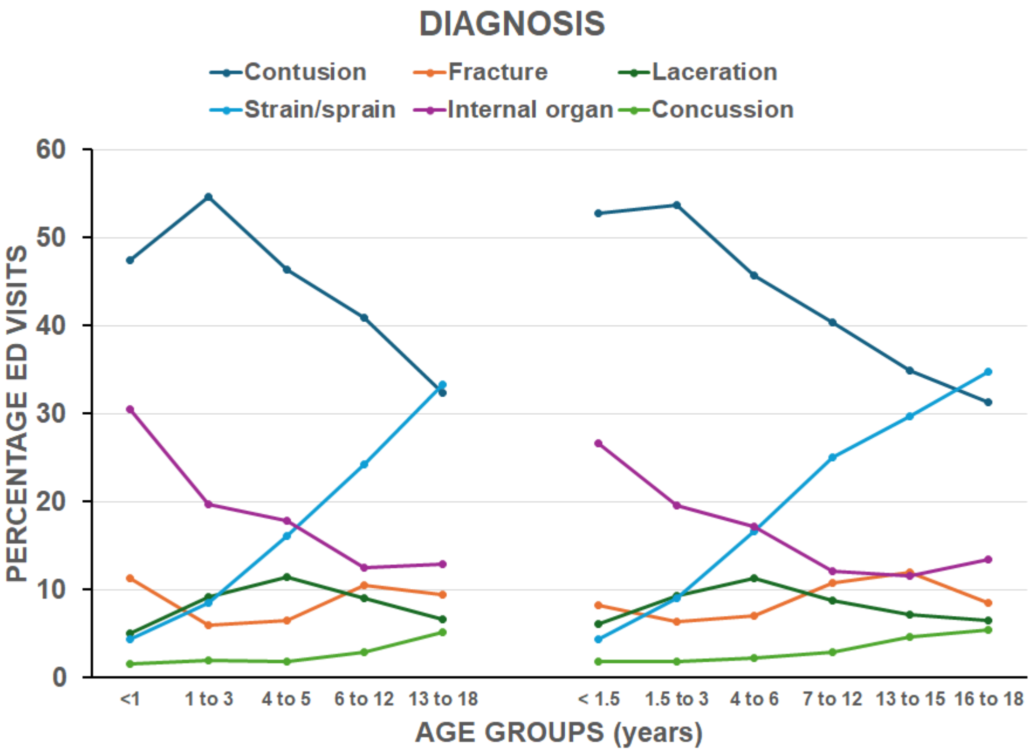

Supplemental figure 1d: Percentage of ED visits by age group ($p < 0.0001$) by ED disposition.

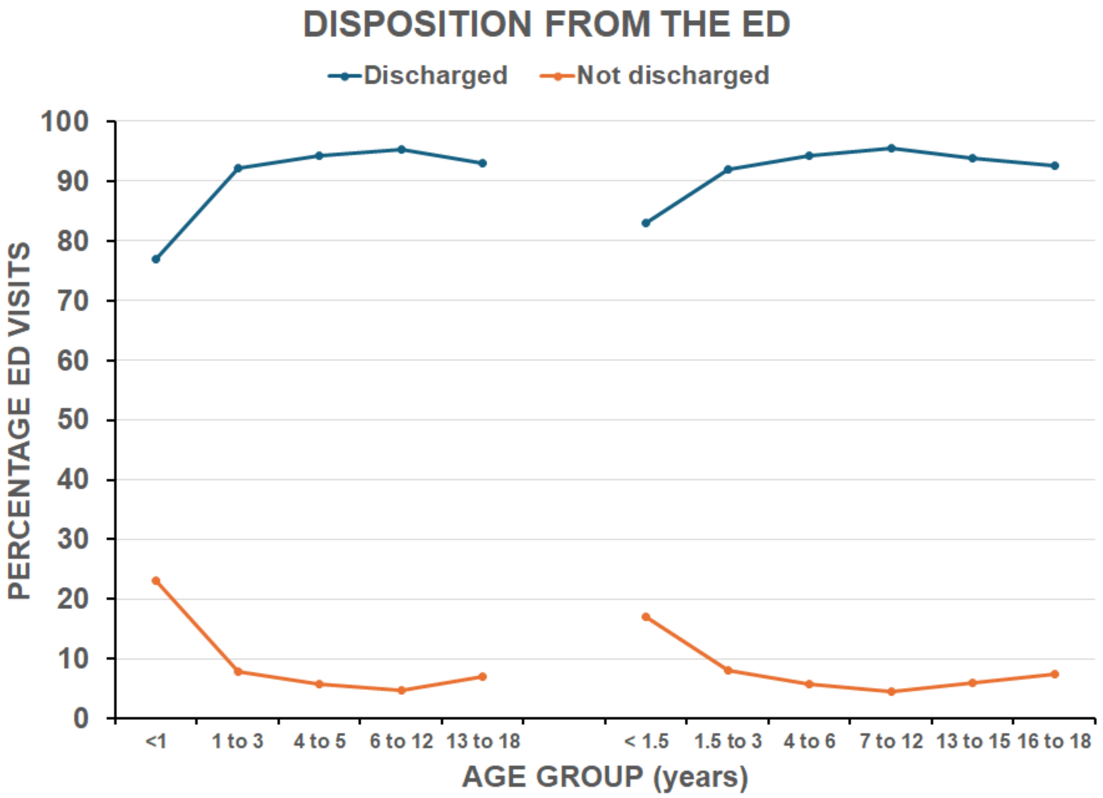

Supplemental figure 1e: Percentage of ED visits by age group ($p < 0.0001$) by anatomic area of injury.

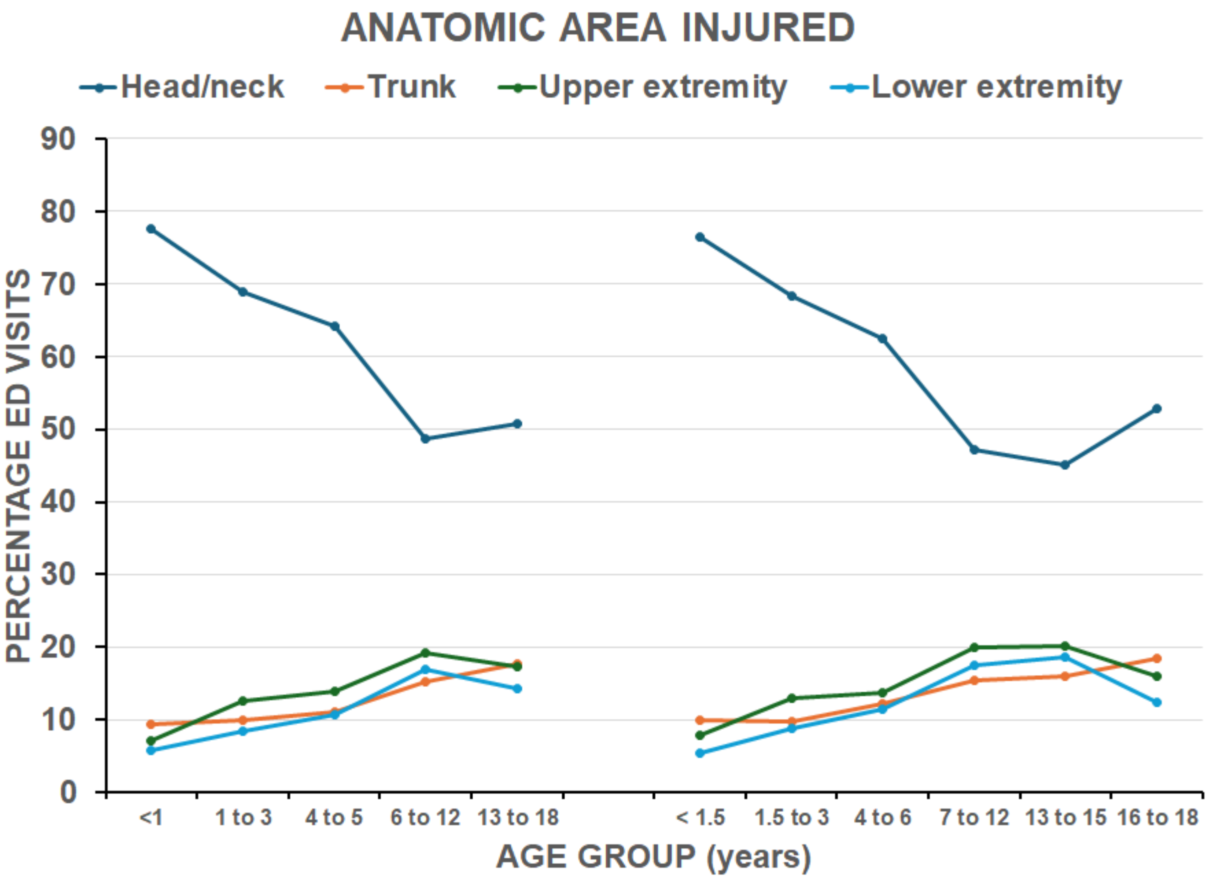

Supplement: Supplementary file 1 [file children-13-00687-s001.zip › supplemental figures.pdf]
